# Functional and quality of life outcomes following obstetric anal sphincter injury (OASI): does the grade of injury affect outcomes?

**DOI:** 10.1007/s00192-017-3334-3

**Published:** 2017-05-18

**Authors:** Lisa Ramage, Clarence Yen, Shengyang Qiu, Constantinos Simillis, Christos Kontovounisios, Paris Tekkis, Emile Tan

**Affiliations:** 1 Department of Surgery and Cancer, Chelsea and Westminster Hospital, Imperial College London NHS Trust, 369 Fulham Road, London, SW10 9NH UK; 2 Department of Colorectal Surgery, The Royal Marsden NHS Foundation Trust, London, UK; 3 Department of Colorectal Surgery, Singapore General Hospital, Bukit Merah, Republic of Singapore

**Keywords:** Obstetric anal sphincter injury, OASI, Functional outcomes, Faecal incontinence

## Abstract

**Introduction and hypothesis:**

The aim of this study was to compare functional and quality of life data in patients with increasing grades of obstetric anal sphincter injury (OASI) presenting to a tertiary colorectal pelvic floor clinic within 24 months of delivery.

**Methods:**

Prospective data were collected from the patients for the period 2009–2016 and included data on functional outcomes and motor anorectal manometry parameters. The instruments used for the evaluation of functional outcomes were the Birmingham Bowel and Urinary Symptoms Questionnaire, the Wexner Incontinence Score, Short Form 36, and the Pelvic Organ Prolapse/Urinary Incontinence Sexual Questionnaire. OASI grade of injury was based on the postdelivery endoanal ultrasound scan. Data from patients with a grade 3a, 3b, 3c or 4 OASI were compared using one-way ANOVA for parametric data and the Kruskal-Wallis test for nonparametric data overall and for separate time periods (3–6 months, 6–12 months, 12–24 months).

**Results:**

Functional patient data were available in 177 patients: 29 with grade 3a, 55 with grade 3b, 77 with grade 3c and 16 with grade 4 OASI. There was no discernible trend in worsening function with increasing severity of OASI overall, nor for the specified time periods of 3–6 months 58 patients), 6–12 months (85 patients) or 12–24 months (18 patients).

**Conclusions:**

Our series demonstrated no significant differences in functional outcomes or quality of life in patients with different OASI grades. Longer-term follow-up is required to ascertain any later functional differences which may become apparent with time.

## Introduction

Obstetric anal sphincter injury (OASI) can be defined as perineal trauma that occurs as a consequence of childbirth and extends to involve part of the anal sphincter complex. It is a recognized cause of faecal incontinence in women [1]. OASI complicates 5.9% of vaginal deliveries in England [2]. The risk is highest in primiparous women, in whom the rate increases to around 6.7%, compared to a rate of 1.7% in multiparous women [3]. Additional risk factors include instrumental delivery, macrosomia (>4 kg birth weight), shoulder dystocia, and Asian ethnicity [2, 4]. Classification of the degree of injury is based upon the degree of anal sphincter involvement; this is an extension of a grading system that encompasses all perineal tears [5]. Grade 1 injuries are limited to the vaginal mucosa or perineal skin, whereas a grade 2 injury extends into the perineal muscles, but is still outwith the anal sphincter complex. Grades 3 and 4 injuries include those involving the anal sphincter. Grade 3a involves up to 50% of the external anal sphincter (EAS). Grade 3b involves up to 100% of the EAS without involvement of the internal anal sphincter (IAS). Grade 3c also involves the IAS, without anal mucosal involvement. In the most severe OASI, grade 4, the injury extends completely through the anal sphincter and the rectal mucosal surface. Whilst the majority of OASI are recognized at the time of injury and undergo primary repair, these injuries can have significant implications on future anal continence. The vast majority of patients experience at least some symptoms in the initial follow-up period, with improvements in function seen over the first year [6]. However, the function of the anal sphincter generally tends to decline with time, with as many as 25% of women with grade 4 injuries experiencing future anal incontinence [7].

Our aim was to assess whether increasing severity of OASI has any impact on function, quality of life (QoL), anorectal manometry (ARM) and sexual function during early stages of follow-up (<24 months) following delivery.

## Materials and methods

This was a retrospective observational study of a prospectively maintained research database with full ethical approval. All included patients were reviewed in a colorectal pelvic floor clinic between 2009 and 2016 inclusive.

According to local trust policy, all women who sustained a third or fourth degree OASI during labour were automatically followed up at an obstetric-led perineal tear clinic. During the consultation, all patients were offered advice on pelvic floor exercises (PFE) and underwent a physical assessment which assessed the degree of perineal wound healing, urinary and bowel continence, any evidence of vaginal prolapse and also the degree of anal rectal tone. If there were concerns regarding the presence of any bowel-related symptoms such as faecal urgency, flatal or faecal incontinence, or if the woman wished to discuss options surrounding future mode of delivery, then referral to a specialist colorectal pelvic floor clinic was arranged.

Initial assessment at the colorectal pelvic floor clinic included a review of obstetric birth history and assessment of current symptoms through the use of several validated patient self-reported medical questionnaires. Patients also underwent formal endoanal ultrasonography (EUS) and ARM studies. Patients with severe symptoms not manageable by conservative PFE were sent for formal biofeedback physiotherapy and followed up every 6–12 months thereafter. If significant symptoms persisted despite biofeedback therapy, secondary sphincter repair or sacral nerve stimulation was subsequently offered. With the clinicopathological information collected at the pelvic floor clinic, a prospectively maintained database was established.

From this database, all patients who presented between 2009 and 2016 and received formal postnatal EUS grading were included in this analysis. Data identified for comparison in this study were recorded prior to any intervention with the exception of primary sphincter repair at the time of delivery and simple PFEs, which had been instigated whilst under gynaecological review. Patients with only follow-up data after treatment intervention were excluded from this analysis. Patients with missed or occult anal sphincter injury who had therefore not undergone primary repair were also excluded. After applying these criteria, 177 consultations were generated from 161 patients.

### EUS and ARM

All woman included in the study underwent EUS either in the colorectal pelvic floor clinic or in the radiology department. EUS findings were used to categorize the final grade of tear according to the recognized OASI grading definitions as discussed above. Additionally, ARM was performed in the pelvic floor clinic as part of quantitative assessment of the motor and sensory function of the anal canal. This was performed using T-DOC® Air-Charged™ ARM catheters with the patient in the left lateral position. Although various motor and sensory assessments were performed, this paper primarily considers sphincter motor function through comparisons of maximum squeeze pressure, maximum resting pressure and squeeze increment. Each reading was taken three times, and the mean of these measurements was used for each of the three parameters in the final analysis.

### Patient-reported outcome measures

The results from four patient-reported outcome measure questionnaires were included in the analysis: the Birmingham Bowel and Urinary Symptoms Questionnaire (BBUSQ), the Wexner Incontinence Score (WIS), the Pelvic Organ Prolapse/Urinary Incontinence Sexual Questionnaire (PISQ-12) and the Short Form 36 (SF-36).

The BBUSQ-22 has frequently been used in the field of pelvic floor dysfunction and includes 22 detailed questions exploring four domains: faecal incontinence, urinary symptoms, evacuatory function and constipation [8]. Each domain is scored out of a 100, with a higher score indicating a higher frequency and severity of symptoms. The WIS is a widely used assessment tool which reports the incidence and nature of anal incontinence. It consists of five questions scored from 0 to 4 depending on the frequency of symptoms, with 0 indicating having “Never” experienced such symptoms and a score of 4 signifying “Always” (more than once per day) [9]. The scores of each domain are added up with 20 points indicating complete incontinence and 0 indicating perfect continence. The PISQ-12 was designed specifically to evaluate sexual function in woman with pelvic floor prolapse. The questions are scored from 0 to 4 with a maximum score of 48, which indicates normal sexual function [10]. The SF-36 score has been used in numerous chronic diseases to measure the overall QoL. This scoring system divides QoL measurements into eight domains with 100% indicating perfect QoL [11]. Each domain is considered separately.

Results from the four questionnaires were analysed as continuous variables, and missing data were handled according to the respective scoring guidelines. The primary outcome was to explore differences in the above parameters according to the final grade of tear as defined by the EUS findings. Data were analysed across all 161 data entries as well as within each of three distinct time periods (3–6 months, 6–12 months and 12–24 months) to limit time as a possible confounder. The secondary outcomes were the prevalence of flatal incontinence, faecal incontinence and faecal urgency according to grade of OASI. These data were obtained from the WIS responses, which quantifies the type of anal incontinence experienced. The data were converted into a binary dataset indicating the presence or absence of symptoms at least once a week. Similarly, question 4 of the BBUSQ which focuses on the symptom of faecal urgency was used, with a score of 3 or 4 (“usually”/“always”) as a positive indicator.

### Statistical analysis

All continuous data were initially tested for normality using normality plots and the Shapiro-Wilk test. Subsequent analysis was performed using one-way ANOVA with the post-hoc Tukey honesty of significance test and the unpaired Student’s *t* test for parametric variables or the Kruskal-Wallis test and the Mann-Whitney *U* test for nonparametric data. The chi-squared test and post-hoc analysis of standard residuals were used to analyse differences in categorical data. All statistical analyses were performed using SPSS version 21.0 with 95% confidence intervals.

## Results

Of the 161 patients accrued from 2009 to 2016, 26 had grade 3a, 52 grade 3b, 71 grade 3c and 12 grade 4 OASI (Table 1). The majority of patients (85) were seen between 6 and 12 months after delivery, and 18 between 12 and 24 months and 58 between 3 and 6 months. At baseline, there were no differences among the patients in terms of age at delivery, parity, the need for episiotomy or an instrumental delivery, history of previous tears and the final OASI grade as defined by EUS. As expected, there were significant differences among the patients seen during the three predefined time periods (*p* < 0.001), validating comparisons made between the datasets.

**Table 1:** Demographic data of the study patients

	Overall	3–6 Months	6–12 Months	12–24 Months	*p* value
No of patients	161	58	85	18	–
Age at delivery (years), mean ± SD	32.9 ± 4.6	32.7 ± 4.2	32.9 ± 5.0	33.7 ± 3.8	0.779
Time to assessment (months), mean ± SD	7.9 ± 4.1	4.6 ± 0.8	8.3 ± 1.5	17.1 ± 3.5	0.000*
Parity, *n* (%)^a^					
1	129 (80.1)	45 (77.6)	70 (82.4)	14 (77.8)	0.546
2	27 (16.8)	9 (15.5)	14 (16.5)	4 (22.2)	
3	4 (2.5)	3 (5.2)	1 (1.1)	0 (0)	
4	1 (0.6)	1 (1.7)	0 (0)	0 (0)	
Episiotomy, *n* (%)^a^	53 (42.1)	17 / 49 (34.7)	30/ 67 (44.8)	6/10 (60.0)	0.258
Instrumental delivery, *n* (%)^a^	81 (62.8)	27/49 (55.1)	45/69 (65.2)	9/11 (81.8)	0.219
Previous tears, *n* (%)^a^	2 (1.3)	0 (0)	2 (2.4)	0 (0)	0.407
Final OASI grade, *n* ^a^					
3A	26	4	20	2	0.082
3B	52	18	26	8	
3C	71	29	34	8	
4	12	7	5	0	

*p < 0.05

^a^ *p* values calculated using the chi-squared test

### Overall analysis

Analysis was performed across all 161 patients regardless of time from delivery across all parameters. Of these patients, 110 (83.3%) reported symptoms of urgency while 16.7%, 30.2% and 6.2% reported symptoms of faecal incontinence, flatal incontinence and combined faecal/flatal incontinence respectively on at least one occasion per week. There were no statistically significant differences in the prevalence of such symptoms across grades of tear (Table 2).

**Table 2:** Prevalence of symptoms of urgency, faecal, flatal and dual incontinence by grade of OASI

Analysis	Symptom	OASI grade								*p* value
		3a		3b		3c		4		
		No. of patients analysed	Percent of patients analysed	No. of patients analysed	Percent of patients analysed	No. of patients analysed	Percent of patients analysed	No. of patients analysed	Percent of patients analysed	
Overall	Urgency	23	73.9	45	84.4	53	84.9	11	90.9	0.560
	Faecal incontinence	17	29.4	35	14.3	35	11.4	9	22.2	0.388
	Flatal incontinence	17	23.5	35	31.4	35	31.4	9	33.3	0.930
	Dual incontinence	17	5.9	35	5.7	35	8.6	9	0.0	0.815
3–6 months	Urgency	4	75	17	82.4	25	92.0	6	100	0.490
	Faecal incontinence	3	0	14	14.3	19	10.5	5	20.0	0.848
	Flatal incontinence	3	0	14	28.6	19	26.3	5	20.0	0.755
	Dual incontinence	3	0	14	7.1	19	10.5	5	0	0.820
6–12 months	Urgency	18	72.2	20	85.0	21	71.4	5	80.0	0.723
	Faecal incontinence	13	38.5	16	6.2	12	0	4	25.0	0.033^a^
	Flatal incontinence	13	30.8	18	37.5	12	33.3	4	50.0	0.91
	Dual incontinence	13	7.7	16	0	12	0	4	0	0.472
12–24 months	Urgency	1	100	8	87.5	7	100	–	–	0.587
	Faecal incontinence	1	0	5	40.0	4	50.0	–	–	0.659
	Flatal incontinence	1	0	5	20.0	4	50	–	–	0.490
	Dual incontinence	1	0	5	20.0	4	25.0	–	–	0.855

^a^The standard residual was +2.4 for grade 3a making this the significant contributor

**Fig. 1:**
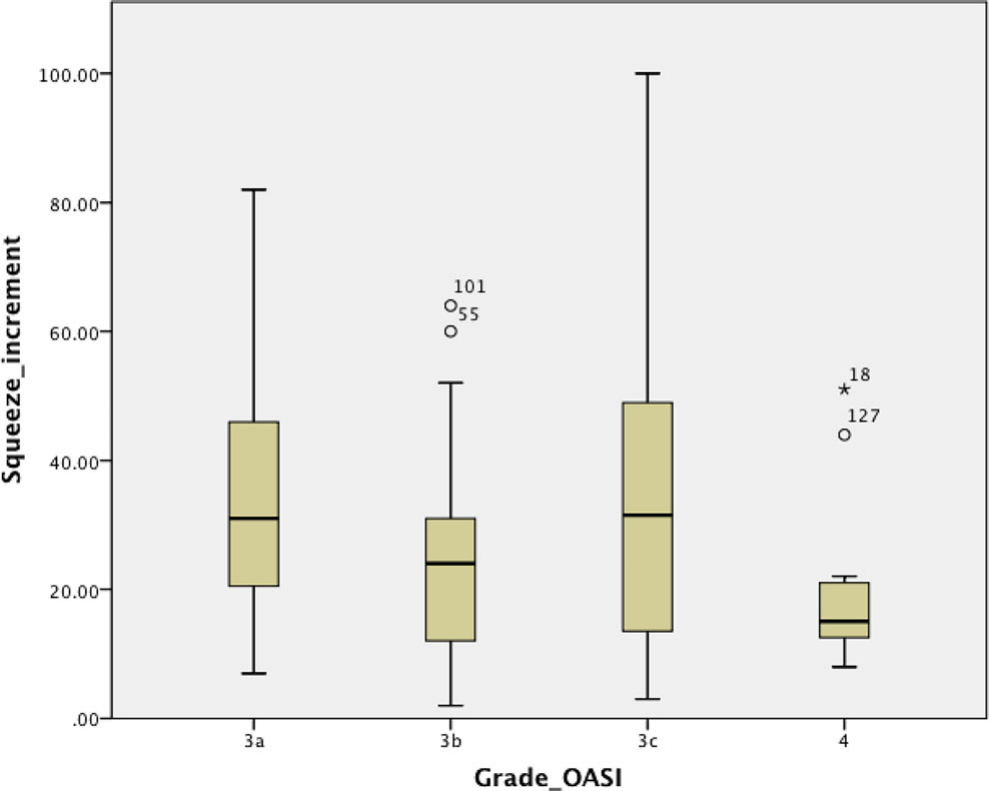
Mean squeeze increment in relation to OASI grade regardless of follow-up interval (*p* = 0.012, grade 3a vs. 3b; *p* = 0.029, grade 3a vs. 4; *p* = 0.015, grade 3b vs. 3c; *p* = 0.017, grade 3c vs. grade 4; ANOVA with unpaired Student’s *t* test)

**Fig. 2:**
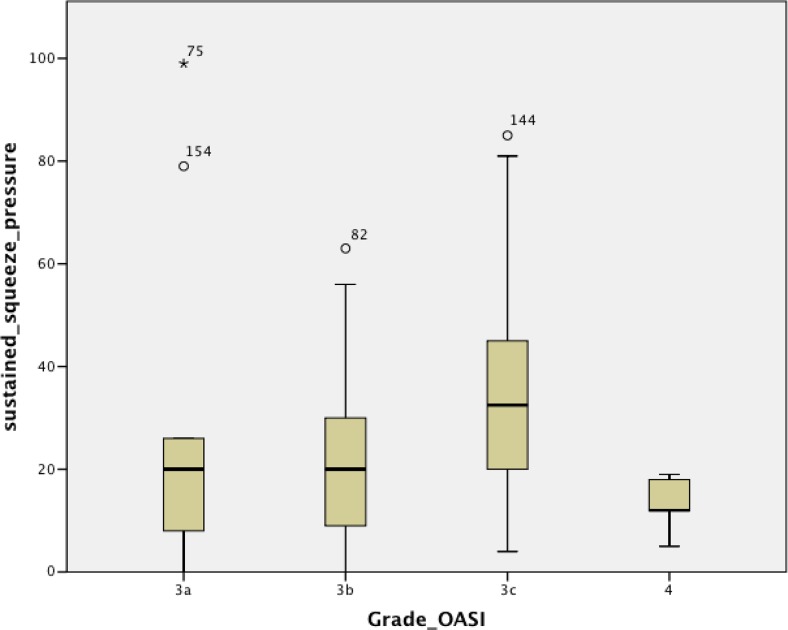
Mean sustained squeeze pressure in relation to OASI grade regardless of follow-up interval (*p* = 0.000, grade 3c vs. grade 4; ANOVA with unpaired Student’s *t* test)

Comparing across all four grades, maximum squeeze increment and the sustained squeeze pressure increment on ARM were the only significantly different parameters. Patients with a grade 3a OASI had a better squeeze increment than those with a grade 3b (*p* = 0.012) or grade 4 (*p* = 0.029) OASI (mean squeeze increments 36.3, 24.5 and 20.5 mm Hg, respectively). Those with a grade 3c OASI had a significantly better squeeze increment than those with a grade 4 OASI (*p* = 0.017; Fig. 1). Patients with a grade 4 OASI had a significantly worse sustained squeeze increment than those with a grade 3c OASI (*p* = 0.000; Fig. 2). The other ARM parameters were not significantly different among the patients with different OASI grades and there were no discernible trends. There were also no significant differences detected across grades amongst the BBUSQ, PISQ-12, Wexner or SF-36 parameters. These results are summarized in Table 3.

**Table 3:** Overall analysis in relation to grade

		OASI grade								*p* value
		3a		3b		3c		4		
		Number of patients	Mean score	Number of patients	Mean score	Number of patients	Mean score	Number of patients	Mean score	
Functional scores	BBUSQ: constipation	22	54.9	44	55.1	61	53.9	8	56.0	0.920
	BBUSQ: incontinence	22	21.2	44	24.7	61	21.42	8	24.5	0.570
	BBUSQ: evacuation	22	11.7	44	15.5	61	12.9	8	6.8	0.089
	BBUSQ: urinary	20	18.0	43	19.11	55	18.6	7	14.0	0.893
	Wexner	19	4.1	33	5.1	44	5.2	7	4.6	0.790
	PISQ-12	8	33.3	22	32.4	31	31.3	7	32.0	0.734
SF-36 quality of life	Physical functioning	21	86.3	38	86.3	49	89.7	4	70.0	0.118
	Role physical	20	88.1	36	84.2	47	86.2	5	83.8	0.611
	Bodily pain	21	69.6	39	81.3	47	84.0	5	82.5	0.075
	General health^a^	21	63.9	37	72.9	43	76.9	5	76.0	0.379
	Vitality^a^	21	52.7	39	51.5	47	52.4	5	52.9	0.995
	Social functioning	21	78.6	38	79.6	47	82.0	5	97.5	0.384
	Role emotional	20	86.7	36	91.7	47	87.1	5	95.0	0.879
	Mental health	21	66.3	39	67.9	52	68.8	7	70.0	0.748
Anorectal manometry	Final resting pressure^a^	22	64.5	43	66.8	60	61.3	12	57.6	0.176
	Final squeeze pressure^a^	22	101.8	41	90.1	60	94.8	11	78.0	0.087
	Maximum squeeze increment	23	36.3	41	24.5	60	33.7	11	20.5	0.030*
	Sustained squeeze increment	9	30.0	17	34.7	30	35.9	5	13.2	0.034*

*p < 0.05

^a^Normally distributed data, therefore ANOVA used

### 3–6 Months

**Fig. 3:**
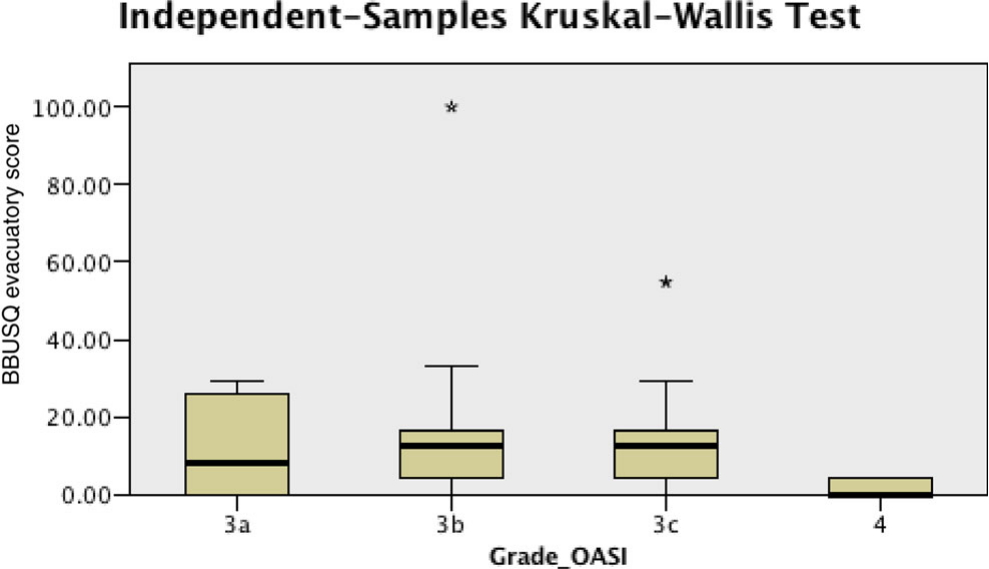
BBUSQ evacuatory scores in relation to OASI grade in patients seen between 3 and 6 months (*p* = 0.040, grade 3b vs. grade 4; *p* = 0.022, grade 3c vs. grade 4; Mann-Whitney *U* test)

Of the 58 patients seen between 3 and 6 months (mean 4.6 ± 0.8 months) after delivery, 88.5% reported faecal urgency, 12.2% reported faecal incontinence, 24.4% reported flatal incontinence and 7.3% reported dual incontinence at least once a week. There was a significant difference in BBUSQ evacuatory scores between patients with a grade 4 OASI and those with a grade 3b or 3c OASI (*p* = 0.040 and *p* = 0.022, respectively; Fig. 3). Patients with a grade 4 OASI had significantly lower evacuatory scores than those with a grade 3b or 3c OASI (1.4 versus 11.5 and 17.4, respectively). None of the other scores of the WIS, BBUSQ, PISQ-12 and SF-36 or the ARM results showed significant differences among the patients with the different OASI grades. These results are summarized in Table 4.

**Table 4:** Functional scores, SF-36 quality of life scores and ARM results in patients seen between 3 and 6 months after delivery

		OASI grade								*p* value
		3a		3b		3c		4		
		Number of patients	Mean score	Number of patients	Mean score	Number of patients	Mean score	Number of patients	Mean score	
Functional scores	BBUSQ: constipation	4	57.5	15	57.6	27	53.5	6	50.8	0.563
	BBUSQ: incontinence^a^	4	22.1	15	24.5	27	22.1	6	18.1	0.302
	BBUSQ: evacuation	4	11.5	15	17.4	27	13.4	6	1.4	0.027*
	BBUSQ: urinary	4	17.5	14	10.9	23	18.3	6	13.9	0.244
	WIS	3	2.7	12	3.9	21	4.4	5	2.8	0.870
	PISQ-12	2	29.0	8	34.3	15	31.75	5	34.6	0.244
SF-36 quality of life	Physical functioning	3	86.7	14	85.0	20	84.0	3	70	0.742
	Role physical	3	79.2	12	81.25	19	76.0	4	82.8	0.870
	Bodily pain	3	58.3	14	81.7	20	76.9	4	90.6	0.436
	General health	3	48.3	12	76.5	18	80.3	4	75	0.224
	Vitality^a^	3	45.8	14	48.1	20	53.8	4	55.2	0.683
	Social functioning	3	70.8	14	77.7	20	79.1	4	100	0.196
	Role emotional	3	75.0	12	97.2	19	84.2	4	93.8	0.237
	Mental health	3	58.3	14	69.6	20	66.4	4	68.8	0.343
Anorectal manometry	Final resting pressure^a^	4	69.7	12	66.8	25	60.1	7	58.4	0.313
	Final squeeze pressure	4	100.7	11	93.8	25	96.0	6	79.5	0.738
	Maximum squeeze increment^a^	4	31.0	11	28.5	25	36.2	6	21.2	0.476
	Sustained squeeze pressure^a^	2	39.5	7	31.4	16	37.1	3	14.3	0.438

*p < 0.05

^a^Normally distributed data, therefore ANOVA used

### 6–12 Months

**Fig. 4:**
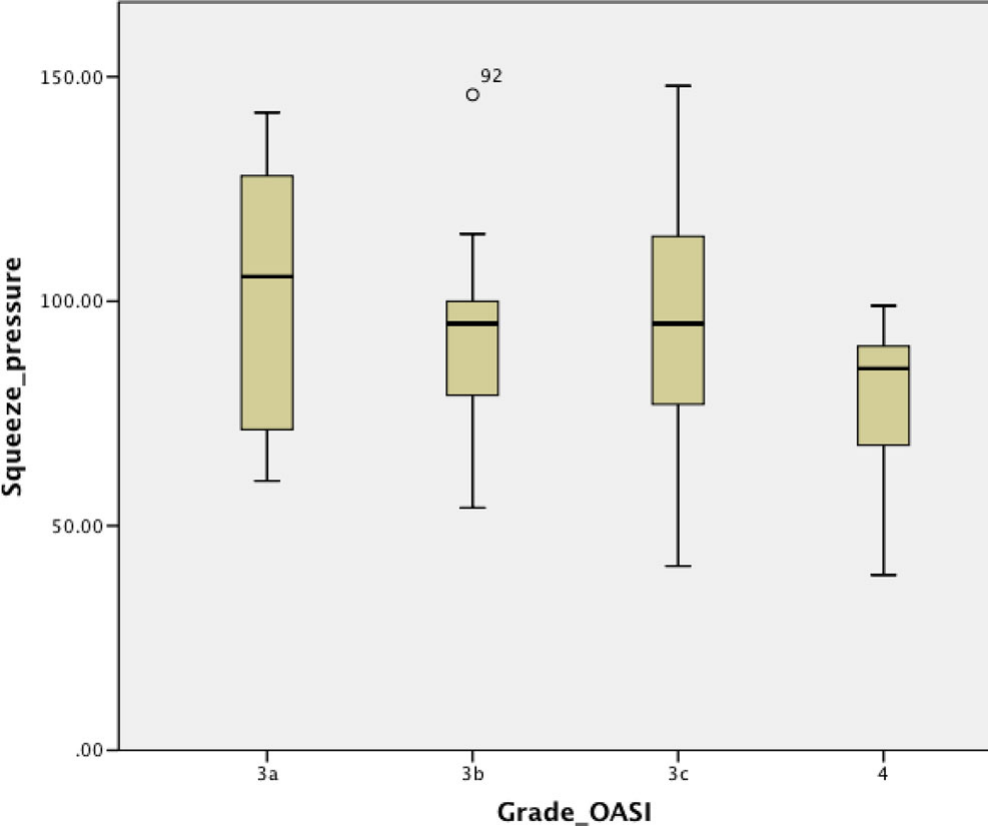
Squeeze pressure in relation to OASI grade in patients seen between 6 and 12 months (*p* = 0.036. grade 3a vs. 3b; *p* = 0029, grade 3b vs. 3c; ANOVA with unpaired Student’s *t* test)

Of 85 patients seen between 6 and 12 months (mean of 8.3 ± 1.5 months) after delivery, 76.6% reported significant faecal urgency, 15.6% reported faecal incontinence, 35.6% reported flatal incontinence and 2.2% reported dual incontinence. Among the 14.3% of patients reporting faecal incontinence there was a statistically significant contribution from the group of patients with a grade 3a OASI with a standard residual of +2.4 in the chi-squared analysis. The scores for the different self-reported scoring instruments were similar across all four grades of OASI (Table 5). The only significant difference detected across the groups was the maximum squeeze increment. Post hoc analysis showed significant differences between patients with a grade 3a and a grade 3b OASI (*p* = 0.036) and between patients with a grade 3b and a grade 3c OASI (*p* = 0.029; Fig. 4).

**Table 5:** Functional scores, SF-36 quality of life questionnaire and ARM results in patients seen between 6 and 12 months after delivery

		OASI grade								*p* value
		3a		3b		3c		4		
		Number of patients	Mean score	Number of patients	Mean score	Number of patients	Mean score	Number of patients	Mean score	
Functional scores	BBUSQ: constipation^a^	16	51.7	21	54.5	27	54.3	2	54.3	0.155
	BBUSQ: incontinence	16	21.1	21	22.03	27	19.3	2	43.7	0.848
	BBUSQ: evacuation	16	11.7	21	16.2	27	13.7	2	23.0	0.608
	BBUSQ: urinary	14	18.2	21	19.9	25	16.7	1	14.3	0.764
	WIS	14	4.0	16	4.5	18	4.1	2	4.5	0.645
	PISQ-12^a^	6	34.7	11	31.3	13	30.1	2	26.5	0.171
SF-36 quality of life	Physical functioning	16	84.8	17	90.3	24	93.8	1	70.0	0.198
	Role physical	15	88.3	17	90.5	23	95.6	1	87.5	0.181
	Bodily pain	16	72.7	18	82.6	22	89.2	1	50	0.520
	General health^a^	16	67.2	18	72.2	21	75.7	1	80	0.591
	Vitality^a^	16	53.5	18	55.0	22	52.8	1	43.8	0.769
	Social functioning	16	82.8	17	86.0	22	86.3	1	87.5	0.725
	Role emotional	15	88.3	19	91.2	26	87.7	1	100	0.875
	Mental health	16	69.2	18	66.2	22	71.1	1	75	0.470
Anorectal manometry	Final resting pressure^a^	16	62.9	25	66.7	27	61.9	5	56.4	0.509
	Final squeeze pressure^a^	16	102.1	25	90.1	27	96.4	5	76.2	0.254
	Maximum squeeze increment^a^	17	37.6	25	23.9	27	34.5	5	19.8	0.042*
	Sustained squeeze pressure	6	15.33	7	19.1	14	34.6	2	11.5	0.138*

*p < 0.05

^a^Normally distributed data, therefore ANOVA used

### 12–24 Months

Of only 18 patients seen between 12 and 24 months (mean 16.9 ± 3.3 months) after delivery, 93.8% reported faecal urgency, 40% reported faecal incontinence, 30% reported flatal incontinence and 20% reported dual incontinence (Table 2). There were no significant differences in functional scores or ARM measurements detected between groups (Table 6).

**Table 6:** Functional scores, SF-36 quality of life questionnaire and ARM results in patients seen between 12 and 24 months after delivery

		OASI grade						*p* value
		3a		3b		3c		
		Number of patients	Mean score	Number of patients	Mean score	Number of patients	Mean score	
Functional scores	BBUSQ: constipation	2	75.0	8	52.1	7	53.8	0.080
	BBUSQ: incontinence	2	16.7	8	32.3	7	26.8	0.453
	BBUSQ: evacuation	2	12.5	8	10.3	7	8.0	0.571
	BBUSQ: urinary	2	17.9	8	31.6	7	26.5	0.309
	WIS	2	6.5	5	10.0	5	5.8	0.360
	PISQ-12	0	–	3	31.7	3	34.3	0.184
SF-36 quality of life	Physical functioning	2	97.5	7	79.2	5	93.0	0.082
	Role physical	2	100	7	74.1	5	82.5	0.117
	Bodily pain	2	62.5	7	76.8	5	67.1	0.376
	General health	2	60.0	7	68.6	4	67.5	0.906
	Vitality	2	56.3	7	49.1	5	45.0	0.403
	Social functioning	2	56.3	7	67.9	5	75.0	0.531
	Role emotional	2	91.7	7	83.3	5	95.0	0.693
	Mental health	2	55.0	7	68.5	5	56.0	0.248
Anorectal manometry	Final resting pressure	2	67.0	6	65.5	8	63.0	0.948
	Final squeeze pressure	2	102.0	5	78.0	8	85.9	0.725
	Maximum squeeze increment	2	35.0	5	19.0	8	22.9	0.694

## Discussion

Overall, our results do not show any major trends for functional decline across increasing OASI grades. With regard to functional scores, only a lower BBUSQ evacuatory score in patients with a grade 4 OASI seen between 3 and 6 months was found to be significant. The overall analysis showed a similar trend, but this was not statistically significant (*p* = 0.089). In all 161 consultations, regular faecal urgency (occurring at least once a week) was present in almost all patients (83.3%), with rates of flatal incontinence, faecal incontinence, and combined flatal and faecal incontinence of 30.2%, 16.7%, and 6.2%, respectively. In contrast, the ARM parameters, in particularly squeeze increment, did demonstrate some significant differences, with evidence of poorer squeeze in patients with a grade 4 OASI than in those with a lower OASI grade. This was seen both in the overall analysis and in the analysis of patients seen between 6 and 12 months.

In the overall analysis squeeze increment was significantly better in patients with a grade 3a OASI than in those with a grade 3b or 4 OASI. Given that there is lesser involvement of the anal sphincter complex in patients with a grade 3a OASI, this result is in agreement with expectation. This result also suggests that maximum squeeze increment is a more sensitive marker than both maximum squeeze pressure and resting pressure. More importantly, there was a trend for decreasing squeeze increment in patients with increasing grade from 3a to 3b to 4. Patients with a grade 3c OASI, however, had a higher squeeze increment than those with a grade 3b OASI, disrupting the trend. In addition, there was a discernible trend of worsening BBUSQ incontinence scores with increasing OASI grade, but the difference was not statistically significant.

Among the patients seen between 3 and 6 months, the findings were inconclusive with no significant trends. Firstly, whilst we would expect that involvement of the IAS in grade 4 tears would result in worse symptoms including constipation than grade 3b tears, the finding of higher constipation scores in patients with a grade 3a tear has not been reported previously. More importantly, these results need to be analysed with caution because all the respective mean BBUSQ constipation scores for each grade were below 64%. According to the validation criteria [8], 64% was validated as the cut-off score to determine the presence of symptoms of clinical constipation in an individual patient. Secondly, while patients with a grade 3a tear had better WIS scores than those with a grade 4 tear, there seemed to be no trend for increasing severity of symptoms with increasing grade. Patients with a grade 3b tear had higher, although insignificant, WIS scores than those with a grade 3c or 4 tear.

Our findings contradict the results of other studies. For example, Mahony et al. [12] studied 500 women at 3 months following sphincter repair. They found that the presence of a major OASI (those extending to involve the IAS) was significantly associated with the presence of faecal incontinence. The difference in findings may relate to our relatively low number of patients with a grade 4 tear (*n* = 21). Similarly, Nichols et al. found that patients with a grade 4 tear were significantly more likely to report new bowel symptoms following delivery than those with a grade 3 tear [13]. Visscher et al. confirmed these findings: patients with combined EAS and IAS tears had worse function at a mean of 5 years following delivery [14].

However, several other studies have shown minimal differences in functional outcomes in patients with differing grades of OASI. For example, Richter et al. [15], in a study of 343 women, found that at 24 weeks there were no significant differences in faecal and anal incontinence rates between women with grade 3 and grade 4 tears, nor among those with different grade 3 OASI subtypes, with the exception of flatal incontinence, which was significantly higher in women with a grade 4 tear. Roos et al. [16] studied 531 patients with a documented OASI who had undergone primary repair. The mean time to follow up was 9 ± 5.9 weeks. In a per-grade analysis, they found no significant differences in individual symptoms between patients with different grades of tear. However, comparing patients with grades 3a and 3b combined (minor OASI) and those with grades 3c and 4 combined (major OASI), those with a major OASI had significantly worse faecal urgency, flatal incontinence and faecal incontinence to liquid stool than those with a lesser degree of tear.

It should be noted that all patients in this study were assessed at a maximum time after delivery of 24 months. With longer follow up, differences in functional outcomes may have become more apparent. Sangalli et al. [7] studied 177 patients, 129 with grade 3 and 48 with grade 4 OASI. At 13 years follow-up, the rates of anal incontinence were 25% and 11.5% in patients with grade 4 and those with grade 3 OASI, respectively. In a study of the prevalence of faecal incontinence, Bharucha et al. [17] found that the median age of patients at onset of symptoms is 55 years or older, with increasing prevalence with increasing age. They suggested that although many patients who sustain OASI are initially asymptomatic, the effects become apparent after later insults cause further deterioration, such as the increase in general pelvic floor laxity associated with the ageing process. Therefore it is not unreasonable to suggest that although in this study population there was little discernible trend in deterioration with increasing grade, with long-term follow-up the prevalence of faecal and anal incontinence is likely to increase, and the effects of differences between grades of insult to the anal sphincter complex are likely to become far more apparent.

### Limitations

The major limitation of this study was its purely observational nature. Patients were not followed up at a specific time point following delivery, and therefore there was a wide range in time to consultation. However, we accounted for this by performing an initial overall analysis and then separate analyses of patients seen 3–6 months, 6–12 months and 12–24 months after delivery; the mean follow-up times for these groups were also statistically significantly different from one another. There may also have been a degree of selection bias, as women who are asymptomatic are possibly less likely to seek further colorectal consultation than those who are symptomatic, although all women considering further vaginal delivery were encouraged to attend for assessment and discussion regardless of current symptom burden. Additionally, only a very small proportion of the patients were seen during the longest follow-up period, with most patients seen prior to 12 months after delivery. The low number of patients may have been the reason why no significant results whatsoever were detected in the analysis of those seen 12–24 months after delivery.

### Conclusions

Our results show that with a relatively short follow-up, there were no apparent differences in functional, sexual or QoL outcomes across patients with all grades of tear, with the exception of a lower WIS score before 6 months in patients with a grade 3a tear. However, the symptom burden across patients with all OASI grades was generally high, particularly faecal urgency and flatal incontinence. Patents who have sustained OASI need appropriate follow-up, aggressive management of symptoms with specialist biofeedback physiotherapy and clear advice regarding the risk of anal incontinence, particularly if further OASI is sustained, as it is likely that with time, the degree of injury will become more apparent and the symptom burden increase. Patients with dual sphincter involvement may benefit from more sustained and aggressive biofeedback physiotherapy to attempt to counteract the risk of future deterioration. Further work is needed to stratify long-term risk according to grade of tear.
